# Distinct DNA methylation in mother-infant dyads exposed to PM2.5 in pregnancy

**DOI:** 10.1186/s13148-025-01997-8

**Published:** 2025-11-21

**Authors:** Eleanor Klibaner-Schiff, Elisabeth M. Simonin, Abhinav Kaushik, Youn Soo Jung, Xiaoying Zhou, Emma Thompson, R. Sharon Chinthrajah, Mary M. Johnson, Kari C. Nadeau

**Affiliations:** 1 Department of Environmental Health, T.H. Chan School of Public Health, Harvard University, 677 Huntington Ave, Boston, MA 02115 USA; 2 Department of Human Genetics, University of Chicago, 920 E 58th St, Chicago, IL 60637 USA; 3 Sean N. Parker Center for Allergy and Asthma, Stanford University, 750 Welch Rd Ste 114, Stanford, CA 94304 USA

**Keywords:** Prenatal air pollution, Pregnancy, DNA methylation, Maternal–fetal interface, Allergy and asthma

## Abstract

Air pollution exposure during pregnancy increases the risk of allergic and respiratory diseases, yet its epigenetic effects across the maternal–fetal interface remain poorly understood. We conducted a pilot study of 10 mother-infant dyads near Fresno, California, to investigate whether prenatal air pollution exposure is associated with differential DNA methylation (DNAm) in maternal peripheral blood mononuclear cells (PBMCs) and infant cord blood mononuclear cells (CBMCs). Using a custom DNAm array targeting high-value allergy- and asthma-associated loci, we identified 24 CpG sites in maternal PBMCs and 18 in infant CBMCs associated with PM2.5 exposure. Only one CpG site was shared between PBMCs and CBMCs, suggesting largely distinct DNAm responses to PM2.5 in maternal and fetal circulations. Further research is needed to validate these associations and determine their functional implications for immune development and disease risk.

**Graphical abstract:**

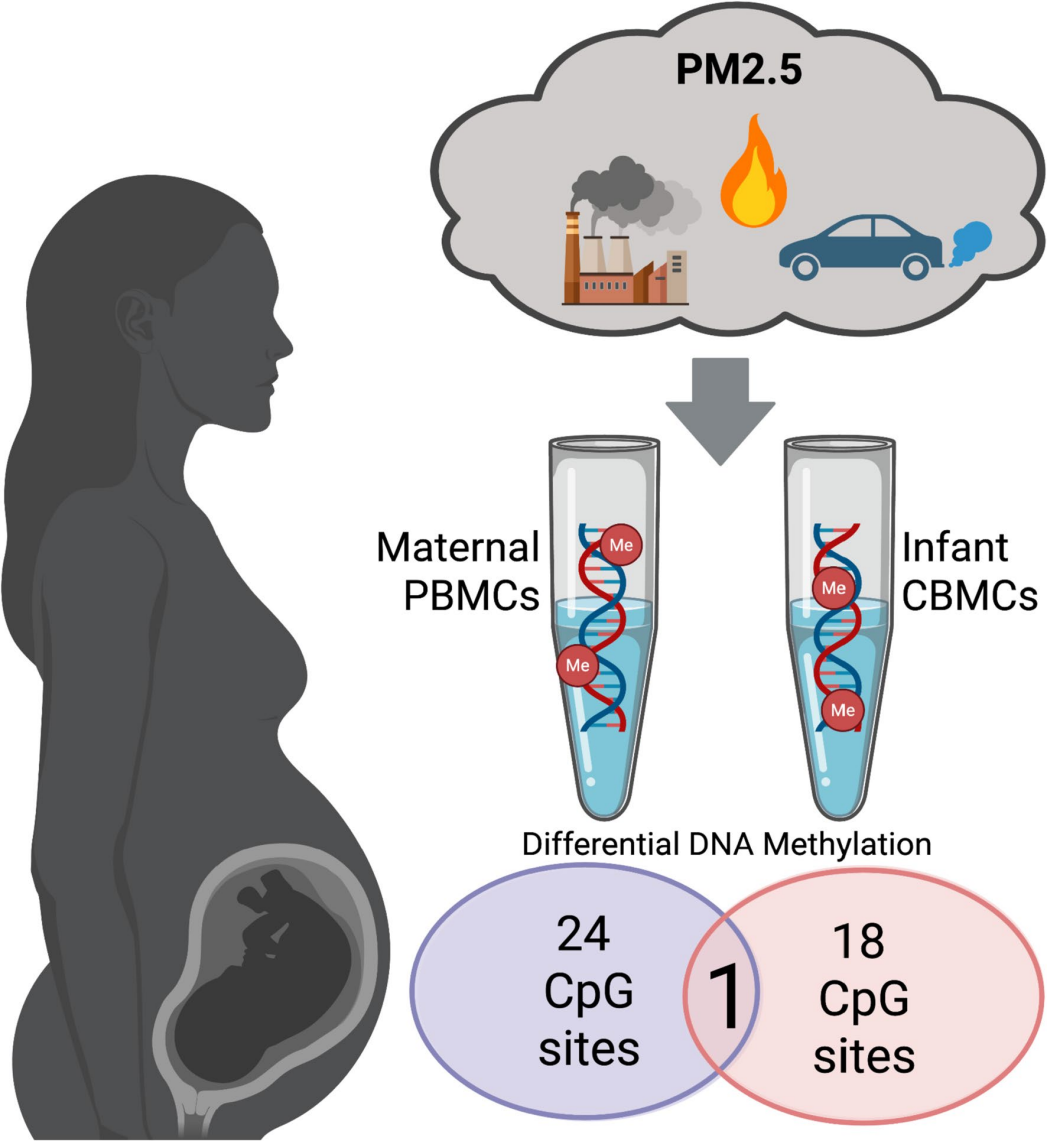

**Supplementary Information:**

The online version contains supplementary material available at 10.1186/s13148-025-01997-8.

## Introduction

Environmental exposures during the prenatal period can shape fetal development and influence long-term health trajectories. Epigenetic modifications, particularly DNA methylation (DNAm), are key mediators of this early-life programming. Dysregulated immune development has been implicated in the rising global incidence of allergic and respiratory conditions, including asthma [4]. Air pollution is a well-established risk factor for the onset and severity of allergy and asthma, and a growing body of literature links exposure to particulate matter under 2.5 microns in diameter (PM2.5) with DNA methylation changes in genes involved in immune signaling and epithelial barrier function [8]. Most previous studies investigating the epigenetic effects of air pollution during pregnancy have focused on either maternal or fetal samples in isolation. In this study, we investigated whether shared environmental exposures during pregnancy produce concordant or distinct DNA methylation patterns in maternal and fetal circulations.

We conducted a pilot study of 10 dyads of pregnant women and newborn infants living near Fresno, California, a region with chronically elevated PM2.5 levels. Using a DNA methylation array targeting high-value asthma- and allergy-related loci, we profiled peripheral blood mononuclear cells (PBMCs) from pregnant women in the second trimester and cord blood mononuclear cells (CBMCs) from their infants at birth. Our goal was to identify CpG sites associated with prenatal PM2.5 exposure in the maternal and infant samples and to assess whether these molecular signals overlapped across the maternal-fetal interface.

## Methods

### Study population

Participants were recruited from the UCSF-Fresno and Clinica Sierra Vista obstetric clinics (*n* = 10). Pregnant women provided a blood sample at their 20 weeks prenatal visit. Cord blood was collected immediately following delivery (*n* = 10). Written, informed permission was obtained from each subject. Enrolled mothers gave written permission for cord blood to be collected at birth. Inclusion criteria were: between 18 and 25 weeks gestation; residence in Fresno, CA or Clovis, CA for a minimum of 3 months prior to enrollment; residence within 20 km of a central air quality monitoring site; no plans to move from the Fresno/Clovis area in the next 2 years; English- or Spanish-speaking; smoked < 50 cigarettes during their pregnancy; and no current or history of cancer, HIV, or autoimmune disease. Exclusion criteria were: oral immunosuppressants within 5 days of the blood draw; history of allergen immunotherapy within 1 year of the visit; chronic disease other than allergies or asthma; and/or current acute infection. All study protocols were approved by Institutional Review Boards at UC Berkeley, UCSF-Fresno, and Stanford University.

### Blood processing, DNA methylation, and air pollution measurement

Whole blood was collected into EDTA tubes and processed within 24 hours. Maternal PBMCs and infant CBMCs were isolated from whole blood using Ficoll-Paque (Cytiva, Marlborough, MA, USA) isolation as previously described [2]. PBMCs and CBMCs were stored in liquid nitrogen until DNA methylation measurement. DNAm was measured using the Allergy&Asthma Custom BeadChip version 1.0 (Illumina) after bisulfite conversion at the University of Chicago Genomics Facility (43,661 probes) [6]. Exclusion criteria for DNAm probes on the Allergy&Asthma array were: underlying single nucleotide polymorphism (SNP); lack of annotated CpG site; lack of annotated genomic coordinates on hg19; location on a sex chromosome. CpG sites at which more than 25% of samples had a DNAm detection *p* value > 0.01 were removed from subsequent analyses. Air pollution data were obtained from the US Environmental Protection Agency’s Air Quality System and interpolated using inverse distance-squared weighting based on the geocoded residential street addresses of participating mothers. Previous research indicates a 3-months exposure window to be most associated with immune and epigenetic changes [8]. Further, our prior work evaluating exposure windows from 1 to 365 days found that 1-year averages showed limited inter-individual variability among participants living near Fresno [8]. For these reasons, average daily PM2.5 exposures were calculated for each mother over a 3-month period prior to blood sample collection.

### Statistical analysis

Systematic quality control was performed on DNAm data using standard analytic pipelines within the R package ‘minfi’ [1]. After data cleaning and quality control, 35,736 CpG sites were analyzed. To evaluate associations between DNAm and PM2.5 exposure, linear regression models were run for each CpG site, using the lm() function in R, with PM2.5 exposure as the predictor variable and DNAm M-values from either maternal or infant samples as the outcome variable. Principal component analysis (PCA) of covariates showed no evidence of confounding by age, eczema status, asthma status, income, or ethnicity in maternal samples, and no evidence of confounding by ethnicity in infant samples (Supplementary Fig. 1). First, a false discovery rate (FDR) correction was applied to each *p*-value calculated from the regressions. As no CpG sites remained significant after FDR adjustment, a dual filter for biological relevance and statistical significance was then applied, retaining CpG sites with an effect size > 0.2 and a nominal *p*-value < 0.05. All statistical analyses and visualizations were performed with R version 4.4.0 in RStudio.

## Results and discussion

Population demographics including air pollution exposure levels are reported in Table 1. The mean age of mothers was 26.5 years (standard deviation: 6.93 years). The majority of enrolled subjects were Hispanic (7/10). Average daily PM2.5 exposures were calculated for each mother over a 3-month period prior to blood sample collection and ranged from 8.6 to 19.3 µg/m^3^.

**Table 1:** Demographics table

Characteristics	Pregnant mothers	Cord blood samples (infants at birth)
	(second trimester)	
Sample size (n)	10	10
Age (years, mean ± SD)	26.5 ± 6.93	0.0 ± 0.0
PM2.5 exposure (ug/m^3^, mean ± SD)	14.14 ± 3.70	n/a
Eczema diagnosis (n)	3	n/a
Food allergies (n)	1	n/a
Asthma diagnosis (n)	5	n/a
Annual income (n)		n/a
Less than $15 k	5	
$15 k—$30 k	3	
$31 k—$50 k	1	
$51 k—$75 k	1	
Ethnicity (n)		
Hispanic	7	6
non-Hispanic Black	1	1
non-Hispanic White	1	1
non-Hispanic Asian	1	1
Multiracial	0	1
Sex (n)		
Female	10	5
Male	0	5

**Fig. 1:**
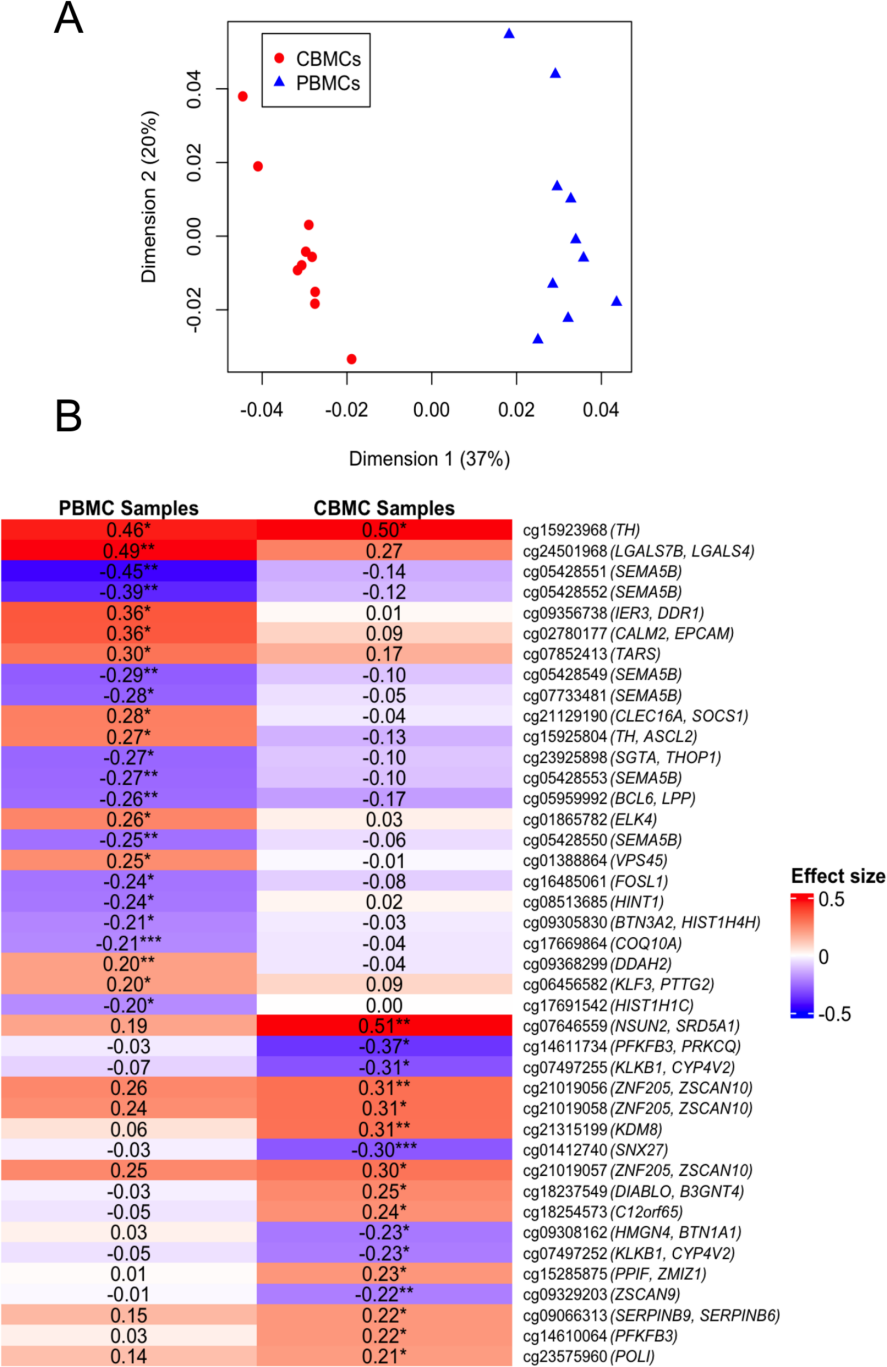
Distinct DNA methylation patterns in pregnant women and infants associated with PM2.5 exposure. **A** Multidimensional scaling analysis of methylation profiles showing separation between maternal PBMCs (blue) and infant CBMCs (red). **B** Heatmap of CpG sites associated with PM2.5 exposure (effect size > 0.2, *p *< 0.05), displaying distinct patterns between maternal and infant samples. Each row denotes a separate CpG site and is annotated with the CpG name and nearest gene(s) in parentheses. (*p < 0.05, **p < 0.01, ***p < 0.001)

Multidimensional scaling analysis of all 35,736 CpG sites showed separate clusters of maternal and infant samples (Fig. 1A). To evaluate associations between DNAm and PM2.5 exposure, linear regression models were run for each CpG site with PM2.5 exposure as the predictor variable and DNAm M-values from either maternal or infant samples as the outcome variable. Given the large number of CpG sites tested and the limited sample size, no CpG sites passed a false discovery rate threshold. We instead applied a dual filter for biological relevance and statistical significance, retaining CpG sites with an effect size > 0.2 and a nominal *p*-value < 0.05, recognizing that results should be interpreted with caution given our small sample. Using this threshold, we identified 24 CpG sites in maternal PBMCs and 18 CpG sites in infant CBMCs associated with PM2.5 exposure (Fig. 1B). One CpG showed significant association with PM2.5 in both maternal PBMCs and infant CBMCs.

We then associated each CpG with its nearest gene transcription start sites using the GREAT (Genomic Regions Enrichment of Annotations Tool) tool [5, 9]. We overlapped each CpG with six functional features, including transcription factor binding sites, transcriptional start sites, active and poised enhancer elements, chromatin accessibility, and chromatin interactions [6]. We report the nearest gene annotations and functional feature annotations for CpG sites associated with PM2.5 exposure in pregnant PBMCs and infant CBMCs in Supplementary Table 1. *TH*, the only gene associated with PM2.5 exposure in both maternal PBMCs and infant CBMCs, encodes tyrosine hydroxylase, an enzyme important for healthy nervous system functioning. In rodent models, PM2.5 exposure has been shown to suppress tyrosine hydroxylase expression [3]. In infant CBMCs, CpG sites associated with prenatal exposure to PM2.5 map to a set of genes involved in neuroendocrine signaling (*TH, CYP4V2*), protease inhibition (*SERPINB6, SERPINB9*), and transcriptional control (*KLF3, ELK4, ZSCAN9*). This suggests that PM2.5 may shape multiple aspects of immune and developmental pathways in utero. Notably, cord blood DNAm at two of these genes, *SERPINB6* and *CYP4V2*, has previously been associated with prenatal NO_2_ exposure, suggesting these loci may serve as common epigenetic targets of air pollution [7]. Among the largely distinct set of CpG sites we found to be associated with PM2.5 exposure in maternal PBMCs, several are near genes involved in immune regulation (*SEMA5B*, *SOCS1, BCL6*) and oxidative stress response (*IER3*).

This study has several limitations. Most notably, the small sample size of *n* = 10 dyads limits statistical power, making it difficult to distinguish consistent exposure-related patterns from inter-individual variation, particularly given the large number of CpG sites tested. While we applied a dual threshold for biological relevance and nominal significance, these results should be interpreted as preliminary and hypothesis-generating rather than conclusive. Finally, although PCA suggested minimal confounding by measured covariates, we acknowledge the potential for unmeasured confounding by sociodemographic factors that may correlate with both PM2.5 exposure and DNAm.

Validation in larger populations of mother-infant dyads will be essential to determine the robustness and generalizability of the associations and patterns identified here. Sex-stratified analyses were not performed due to limited sample size; future, larger studies should test for sex-specific effects of PM2.5 exposure across the maternal–fetal interface. Although we focused on 3-month exposures in this pilot study, later work in larger and more diverse populations should examine long-term exposures across pregnancy. Future studies may also investigate whether air pollution is associated not only with mean differences in DNA methylation, but also with variability across individuals, which will require larger sample sizes.

In conclusion, this study leverages a specialized DNA methylation array targeting high-value allergy- and asthma-associated loci to provide early evidence that PM2.5 exposure during pregnancy is associated with differential DNAm in both maternal PBMCs and infant CBMCs, with largely distinct effects in the two sample types. These findings suggest that fetal DNA methylation patterns may be modified in utero, and that PM2.5 exposure may exert distinct effects across the placenta in maternal and fetal circulations. Further research with larger cohorts and integrated multi-omic analyses is needed to validate these findings and characterize their functional consequences. As air pollution exposures rise globally, this work contributes to our understanding of immune vulnerabilities during pregnancy and how in utero environmental exposures may shape fetal immune development.

## Supplementary Information

Additional file1

## Data Availability

The datasets analyzed in the current study are available from the study team upon reasonable request.

## Abbreviations

CBMCs: Cord blood mononuclear cells
DNAm: DNA methylation
FDR: False discovery rate
PCA: Principal component analysis
PM2.5: Particulate matter under 2.5 microns in diameter
PBMCs: Peripheral blood mononuclear cells
SNP: Single nucleotide polymorphism
