# Supplementary material for: Distinct DNA methylation in mother-infant dyads exposed to PM2.5 in pregnancy
Source: Clin Epigenetics. 2025 Nov 21;17:197. doi: 10.1186/s13148-025-01997-8 (PMC12639737; doi:10.1186/s13148-025-01997-8)
Supplement: Supplementary file 1 — Additional file1 [file 13148_2025_1997_MOESM1_ESM.docx]


**Supplementary Figure 1. No significant correlation was found between potential covariates and DNAm principal components in maternal PBMCs or infant CBMCs.** (A) Pearson correlation coefficients between ethnicity, age, eczema status, asthma status, income and the first four DNAm principal components in maternal PBMCs. No correlation had a p-value < 0.05. (B) Pearson correlation coefficients between ethnicity and the first four DNAm principal components in infant CBMCs. No correlation had a p-value < 0.05.

**Supplementary Table 1.** Associated genes nearest the CpG sites with significant association with PM2.5 exposure in maternal PBMCs (24 CpGs) or infant CBMCs (18 CpGs).

| **Cell Source** | **CpG** | **Associated Gene(s)^1^** | **Functional Annotations** |
| --- | --- | --- | --- |
| Maternal PBMCs | cg01388864 | VPS45 | TFBS, ATAC, HiC Capture, TSS |
| Maternal PBMCs | cg01865782 | ELK4 | TFBS, HiC Capture, Active Enhancer |
| Maternal PBMCs | cg02780177 | CALM2, EPCAM | TFBS, ATAC, HiC Capture, Active Enhancer |
| Maternal PBMCs | cg05428549 | SEMA5B | TFBS, ATAC, HiC Capture, Active Enhancer |
| Maternal PBMCs | cg05428550 | SEMA5B | TFBS, ATAC, HiC Capture, Active Enhancer |
| Maternal PBMCs | cg05428551 | SEMA5B | TFBS, ATAC, HiC Capture, Active Enhancer |
| Maternal PBMCs | cg05428552 | SEMA5B | TFBS, ATAC, HiC Capture, Active Enhancer |
| Maternal PBMCs | cg05428553 | SEMA5B | TFBS, HiC Capture, Active Enhancer |
| Maternal PBMCs | cg05959992 | BCL6, LPP | TFBS, ATAC, HiC Capture, TSS |
| Maternal PBMCs | cg06456582 | KLF3, PTTG2 | TFBS, HiC Capture, Poised Enhancer, Active Enhancer |
| Maternal PBMCs | cg07733481 | SEMA5B | TFBS, ATAC, HiC Capture, Active Enhancer |
| Maternal PBMCs | cg07852413 | TARS | TFBS, HiC Capture, TSS |
| Maternal PBMCs | cg08513685 | HINT1 | TFBS, ATAC, HiC Capture, TSS |
| Maternal PBMCs | cg09305830 | BTN3A2, HIST1H4H | TFBS, HiC Capture, TSS, Active Enhancer |
| Maternal PBMCs | cg09356738 | IER3, DDR1 | TFBS, HiC Capture, Active Enhancer |
| Maternal PBMCs | cg09368299 | DDAH2 | TFBS, ATAC, HiC Capture, TSS, Poised Enhancer |
| Maternal PBMCs, Infant CBMCs | cg15923968 | TH | TFBS, ATAC, Active Enhancer |
| Maternal PBMCs | cg15925804 | TH, ASCL2 | TFBS, ATAC, HiC Capture, TSS |
| Maternal PBMCs | cg16485061 | FOSL1 | TFBS, ATAC, HiC Capture, TSS |
| Maternal PBMCs | cg17669864 | COQ10A | TFBS, ATAC, HiC Capture, TSS |
| Maternal PBMCs | cg17691542 | HIST1H1C | TFBS, ATAC, HiC Capture, TSS |
| Maternal PBMCs | cg21129190 | CLEC16A, SOCS1 | TFBS, ATAC, HiC Capture, Poised Enhancer, Active Enhancer |
| Maternal PBMCs | cg23925898 | SGTA, THOP1 | TFBS, ATAC, HiC Capture |
| Maternal PBMCs | cg24501968 | LGALS7B, LGALS4 | TFBS, ATAC, HiC Capture, Active Enhancer |
| Infant CBMCs | cg01412740 | SNX27 | TFBS, ATAC, HiC Capture, TSS |
| Infant CBMCs | cg07497252 | KLKB1, CYP4V2 | TFBS, ATAC, HiC Capture, TSS, Active Enhancer |
| Infant CBMCs | cg07497255 | KLKB1, CYP4V2 | TFBS, ATAC, HiC Capture, TSS, Active Enhancer |
| Infant CBMCs | cg07646559 | NSUN2, SRD5A1 | TFBS, ATAC, Active Enhancer |
| Infant CBMCs | cg09066313 | SERPINB9, SERPINB6 | TFBS, ATAC, HiC Capture, Poised Enhancer, Active Enhancer |
| Infant CBMCs | cg09308162 | HMGN4, BTN1A1 | TFBS, ATAC, HiC Capture, TSS, Active Enhancer |
| Infant CBMCs | cg09329203 | ZSCAN9 | TFBS, ATAC, HiC Capture, TSS |
| Infant CBMCs | cg14610064 | PFKFB3 | TFBS, ATAC, HiC Capture, TSS |
| Infant CBMCs | cg14611734 | PFKFB3, PRKCQ | TFBS, Active Enhancer |
| Infant CBMCs | cg15285875 | PPIF, ZMIZ1 | TFBS, HiC Capture, Poised Enhancer, Active Enhancer |
| Infant CBMCs | cg18237549 | DIABLO, B3GNT4 | TFBS, ATAC, HiC Capture, TSS |
| Infant CBMCs | cg18254573 | C12orf65 | TFBS, ATAC, HiC Capture, TSS |
| Infant CBMCs | cg21019056 | ZNF205, ZSCAN10 | TFBS, TSS, Active Enhancer |
| Infant CBMCs | cg21019057 | ZNF205, ZSCAN10 | TFBS, TSS, Active Enhancer |
| Infant CBMCs | cg21019058 | ZNF205, ZSCAN10 | TFBS, TSS, Active Enhancer |
| Infant CBMCs | cg21315199 | KDM8 | TFBS, ATAC, HiC Capture, TSS |
| Infant CBMCs | cg23575960 | POLI | TFBS, ATAC, HiC Capture, TSS |

1. Associated genes were derived from the GREAT tool. Functional annotations were shown by Morin et al. (2023) in the development of the Allergy&Asthma DNAm array.
